# A36 INVESTIGATING TYPE 1 REGULATORY T CELLS AS A THERAPY FOR INFLAMMATORY BOWEL DISEASE USING A MOUSE MODEL OF ACUTE INTESTINAL EPITHELIAL DAMAGE

**DOI:** 10.1093/jcag/gwac036.036

**Published:** 2023-03-07

**Authors:** C Poloni, A Sze, X Wang, S Lim, T Steiner

**Affiliations:** 1 Microbiology & Immunology, University of British Columbia; 2 BC Children's Hospital Research Institute, Vanccouver; 3 BC Children's Hospital Research Institute, Vancouver, Canada

## Abstract

**Background:**

Inflammatory bowel disease (IBD) affects an estimated 270,000 people in Canada and is rapidly increasing in prevalence. All patients have relapsing disease, and a subset of individuals do not respond to current treatments. Further, there are no approved treatment options in Canada that reverse IBD-induced intestinal fibrosis. We have previously shown type 1 regulatory cells (Tr1s) are capable of suppressing inflammatory macrophages, promote barrier function of human intestinal epithelial cells, and induce differentiation of mucin-producing goblet cells. We hypothesize that Tr1 cells can prevent inflammatory damage and fibrosis in an mouse model of acute gut damage.

**Purpose:**

We hypothesize that Tr1 cells can prevent inflammatory damage and fibrosis in an mouse model of gut damage. Here we evalute the therapeutic potential of Tr1 cells in an model of acute intestinal epithelial damage.

**Method:**

Tr1 cells were isolated and expanded from CD4^+^ CD44^high^ FOXP3^-^ cells. Their phenotype was characterized by flow cytometry and cytokine secretion was measured *via* ELISA. WT B6 mice were given 2% DSS in H_2_O for 7 days, followed by H_2_O alone for 7 days. Prior to DSS treatment, mice were sub-lethally irradiated to facilitate engraftment, and given I.P. injections of PBS or 0.5 – 2 x 10^6^ Tr1 cells. Mice weights and health scores were recorded daily. At the endpoint, blood, spleen, and mesenteric lymph nodes were analyzed for Tr1 cell engraftment (or lack thereof) for each mouse. Complete white blood counts were performed for each mouse. Additionally, proximal, medial, and distal portions of the ileum were processed for histologic scoring.

**Result(s):**

Tr1 cells isolated from CD4^+^ CD44^high^ FOXP3^-^ cells produce high levels of IL-10 following stimulation (>35,000 pg/ml/1 x 10^5^ cells). Additionally, these cells express high levels of Tr1 markers CD49b and Lag-3. Optimization experiments indicated no significant differences between mice irradiated and given DSS and mice only given DSS (no irradiation). Our results suggest no significant differences in inflammatory cell infiltrate scores between control and Tr1 treated mice. However, gut architecture scores appeared to improve with increasing Tr1 doses. Further, weight change improved with Tr1 treatment, as compared to PBS controls. Interestingly, Tr1 treatment appeared to decrease total eosinophil and neutrophil counts from peripheral blood.

**Conclusion(s):**

Our initial findings indicate Tr1 adoptive transfer prior to acute damage via DSS improves gut damage and weight loss.

**Please acknowledge all funding agencies by checking the applicable boxes below:**

CIHR

**Disclosure of Interest:**

None Declared

